# Inhibitors of Human 5-Lipoxygenase Potently Interfere With Prostaglandin Transport

**DOI:** 10.3389/fphar.2021.782584

**Published:** 2022-01-21

**Authors:** Astrid S. Kahnt, Carlo Angioni, Tamara Göbel, Bettina Hofmann, Jessica Roos, Svenja D. Steinbrink, Florian Rörsch, Dominique Thomas, Gerd Geisslinger, Kai Zacharowski, Sabine Grösch, Dieter Steinhilber, Thorsten J. Maier

**Affiliations:** ^1^ Institute of Pharmaceutical Chemistry, Goethe University, Frankfurt, Germany; ^2^ Pharmazentrum Frankfurt/ZAFES, Institute of Clinical Pharmacology, Goethe-University, Frankfurt, Germany; ^3^ Paul-Ehrlich Institute, Federal Institute for Vaccines and Biomedicines, Langen, Germany; ^4^ Department of Anesthesiology, Intensive Care Medicine and Pain Therapy, University Hospital Frankfurt, Goethe University, Frankfurt, Germany

**Keywords:** prostaglandin, lipoxygenase inhibitor, multidrug resistance protein 4, inflammation, ABC transporter, eicosanoid

## Abstract

5-Lipoxygenase (5-LO) is the key enzyme in the formation of pro-inflammatory leukotrienes (LT) which play an important role in a number of inflammatory diseases. Accordingly, 5-LO inhibitors are frequently used to study the role of 5-LO and LT in models of inflammation and cancer. Interestingly, the therapeutic efficacy of these inhibitors is highly variable. Here we show that the frequently used 5-LO inhibitors AA-861, BWA4C, C06, CJ-13,610 and the FDA approved compound zileuton as well as the pan-LO inhibitor nordihydroguaiaretic acid interfere with prostaglandin E_2_ (PGE_2_) release into the supernatants of cytokine-stimulated (TNFα/IL-1β) HeLa cervix carcinoma, A549 lung cancer as well as HCA-7 colon carcinoma cells with similar potencies compared to their LT inhibitory activities (IC_50_ values ranging from 0.1–9.1 µM). In addition, AA-861, BWA4C, CJ-13,610 and zileuton concentration-dependently inhibited bacterial lipopolysaccharide triggered prostaglandin (PG) release into human whole blood. Western Blot analysis revealed that inhibition of expression of enzymes involved in PG synthesis was not part of the underlying mechanism. Also, liberation of arachidonic acid which is the substrate for PG synthesis as well as PGH_2_ and PGE_2_ formation were not impaired by the compounds. However, accumulation of intracellular PGE_2_ was found in the inhibitor treated HeLa cells suggesting inhibition of PG export as major mechanism. Further, experiments showed that the PG exporter ATP-binding cassette transporter multidrug resistance protein 4 (MRP-4) is targeted by the inhibitors and may be involved in the 5-LO inhibitor-mediated PGE_2_ inhibition. In conclusion, the pharmacological effects of a number of 5-LO inhibitors are compound-specific and involve the potent inhibition of PGE_2_ export. Results from experimental models on the role of 5-LO in inflammation and pain using 5-LO inhibitors may be misleading and their use as pharmacological tools in experimental models has to be revisited. In addition, 5-LO inhibitors may serve as new scaffolds for the development of potent prostaglandin export inhibitors.

## Introduction

**FIGURE 1:**
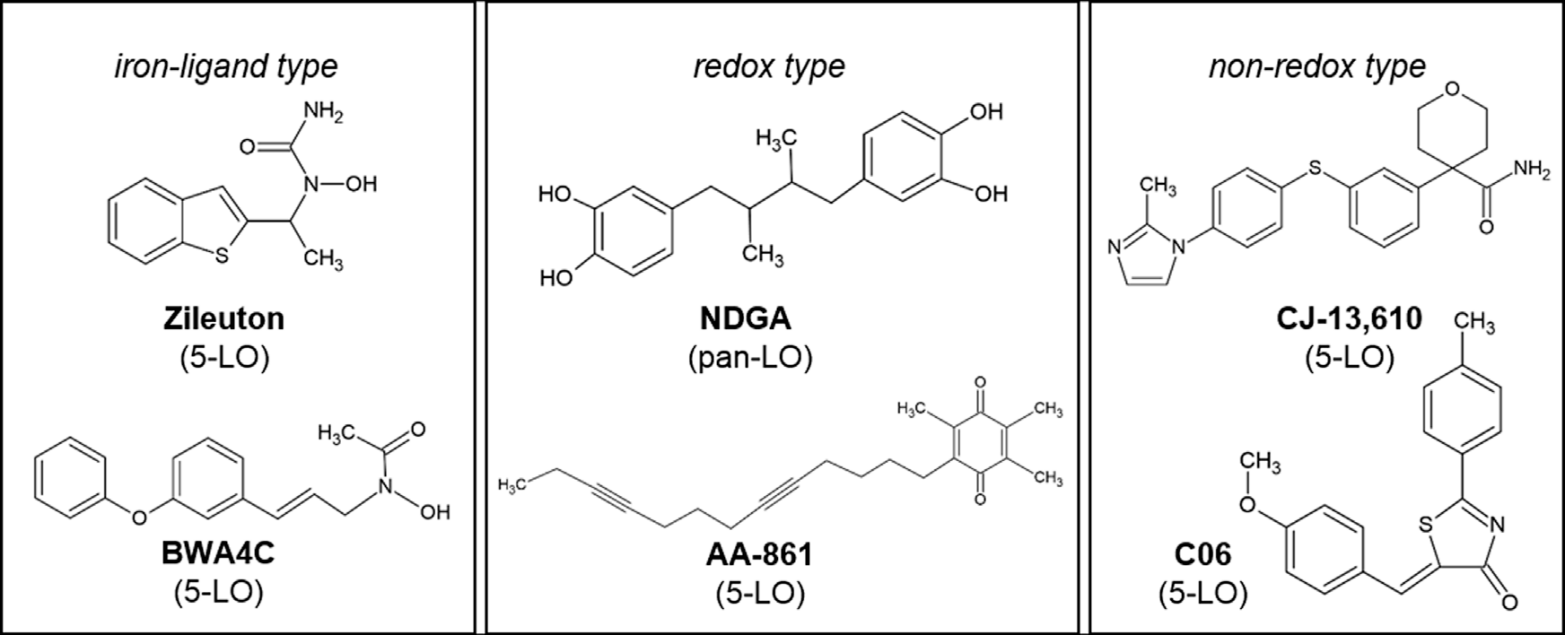
Chemical structures of the 5-LO inhibitors. Inhibitors are grouped according to their postulated mode of action.

The 5-Lipoxygenase (5-LO) inhibitors AA-861, BWA4C, C06, CJ-13,610 and the FDA approved compound zileuton as well as the pan-LO inhibitor nordihydroguaiaretic acid (NDGA) (Figure 1) are frequently used to study the role of 5-LO and leukotrienes (LT) in models of inflammation, pain and cancer. These inhibitors suppress the activity of 5-LO through a number of different modes of action. Iron-ligand inhibitors including zileuton and BWA4C inhibit enzyme activity through chelation of the central iron atom and/or by stabilizing its ferrous state. Non-redox- and redox-type inhibitors including CJ-13,610, AA-861, C06 and NDGA are thought to compete with the substrate arachidonic acid (ARA) for binding to the active site. Due to pharmacokinetic difficulties as well as severe side-effects most of the 5-LO inhibitors failed to gain market authorization in the past. Only zileuton successfully entered the United States market for the treatment of asthma. However, its use is limited due to its short half-life and rare cases of hepatotoxicity (Werz and Steinhilber, 2005; Werz and Steinhilber, 2006; Steinhilber and Hofmann, 2014).

Nevertheless, 5-LO inhibitors are frequently used to evaluate the role of 5-LO and LT in the pathogenesis of various chronic inflammatory diseases as well as cancer and beneficial effects seen with these inhibitors are frequently attributed to the reduction in LT formation. Although all inhibitors effectively suppress LT biosynthesis, the pharmacodynamic profiles and therapeutic effects in these *in vitro* and *in vivo* experiments greatly vary among compounds. In addition, contradictory therapeutic efficacies have been observed (Werz and Steinhilber, 2006; Steinhilber and Hofmann, 2014). Apparently, the pharmacological properties of 5-LO inhibitors are diverse and seem at least partially related to off-target effects which are not fully understood so far. Indeed, in recent years several publications could show that at least some of the anti-inflammatory and anti-carcinogenic properties of these compounds are due to off-target effects (Mancini et al., 2001; Fischer et al., 2010; Rossi et al., 2010).

Like LTs, prostaglandins (PG) are ARA-derived lipid mediators intertwined with numerous physiological and pathophysiological processes, among them the maintenance of homeostatic functions such as blood perfusion and integrity of the gastric mucosa. In addition, PGs play a central role in inflammation contributing to the initiation of pain, swelling, fever and also immune cell coordination thereby triggering the cardinal signs of inflammation. According to this, drugs that inhibit PG formation are effective anti-inflammatory and anti-pyretic therapeutics (Ricciotti and FitzGerald, 2011).

PGE_2_ is the most important PG involved in the pathophysiology of innumerable inflammatory disorders, cancer and pain. Upon an inciting stimulus the production of PGE_2_ is potently upregulated in immune cells by activation as well as elevation of PG pathway enzymes: First, ARA is liberated from membrane phospholipids by cytosolic phospholipase A_2α_ (cPLA_2α_) and then converted into PGH_2_ by one of the two cyclooxygenase isoforms through cyclisation and oxygenation steps. Subsequently, PGH_2_ can be further converted into the prostanoids PGE_2_, TXA_2_, PGD_2_, PGF_2α_ or PGI_2_ by different down-stream synthases depending on the cellular layout and stimulus present (Ricciotti and FitzGerald, 2011).

Since PG and LT share the precursor lipid ARA, we decided to investigate the influence of the 5-LO inhibitors AA861, BWA4C, C06, CJ-13,610, zileuton as well as the pan-LO inhibitor NDGA on prostaglandin formation in several human cell lines as well as human whole blood.

## Materials and Methods

### Drugs, Chemical Reagents and Other Materials

Arachidonic acid, AA-861, aspirin, BWA4C, CHAPS (3-[(3-Cholamidopropyl) dimethylammonio]-1-propanesulfonate hydrate, CJ-13,610, creatine phosphate, DAPI (4′,6-Diamidine-2-phenylindole), dexamethasone, FAF-BSA (fatty acid-free bovine serum albumin), IL-1β, indomethacin, LPS (bacterial lipopolysaccharide), Mowiol 4–88, NDGA (nordihydroguaiaretic acid), PFA (paraformaldehyde), rofecoxib, SC-560 and Tween 20 were purchased from Sigma-Aldrich (Munich, Germany). MF63, PGE_2_, Mk-571 and zileuton were bought from Cayman Chemical (Ann Arbor, MI, United States). FCS (fetal bovine serum) was obtained from Biochrom AG (Berlin, Germany). DMEM (Dulbecco’s modified eagle medium) and HBSS (Hank’s balanced salt solution) were purchased from Gibco/Invitrogen (Paisley, United Kingdom). PBS (phosphate buffered saline), penicillin, streptomycin and sodium pyruvate were bought from PAA laboratory GmbH (Pasching, Austria). The cPLA_2α_ inhibitor, N-{(2S,4R)-4-(Biphenyl-2-ylmethyl-isobutyl-amino)-1-[2-(2,4-difluorobenzoyl)-benzoyl]-pyrrolidin-2-ylmethyl}-3-[4-(2,4-dioxothiazolidin-5-ylidenemethyl)-phenyl]acrylamide HCl (Pyr), was obtained from Calbiochem (Merck KGaA, Darmstadt, Germany). TNFα was purchased from Peprotech GmbH (Hamburg, Germany). PSC-833 was bought from St. Cruz Biotechnology (Heidelberg, Germany). BCECF (2′,7′-Bis(2-carboxyethyl)-5(6)-carboxyfluorescein) and BCECF-AM (BCECF tetrakis(acetoxymethyl) ester) were obtained from Molecular Probes, Inc. (Eugene, OR, United States). TritonX100 was purchased from USB Biochemical (Cleveland, OH, United States). Protease inhibitor cocktail tablets (cOmplete miniTM) and creatine kinase were bought from Roche Diagnostic GmbH (Mannheim, Germany). DMSO (Dimethylsulfoxide), HEPES (4-(2-hydroxyethyl)-1-piperazineethanesulfonic acid), Na_3_PO_4_, SDS (sodium dodecyl sulfate) and TrisHCl (Tris(hydroxymethyl)aminomethane hydrochloride) were from AppliChem GmbH (Darmstadt, Germany). EDTA (Ethylenediaminetetraacetic acid) sucrose and MgCl_2_ were bought from Merck KGaA (Darmstadt, Germany). ATP (adenosine triphosphate) was bought from Carl Roth GmbH (Karlsruhe, Germany).

### Cell Culture

The human cervix carcinoma cell line HeLa as well as A549 lung cancer cells were purchased from Deutsche Sammlung für Mikroorganismen und Zellkulturen (DSMZ, Braunschweig, Germany). HCA-7 colon carcinoma cells were obtained from the European Collection of Cell Cultures (ECACC, Salisbury, United Kingdom). HEK293/4.63 overexpressing MRP-4 cells (2) were kindly provided by Professor Dr. Piet Borst (Netherlands Cancer Institute, Amsterdam, Netherlands). All cell lines were maintained in DMEM supplemented with 10% fetal calf serum (FCS), 1 mM sodium pyruvate, 100 μg mL^−1^ streptomycin and 100 U•mL^−1^ penicillin (complete growth medium). Cells were kept at 37°C in a humidified atmosphere containing 5% CO_2_.

### Stimulation of Prostaglandin Formation in Cell Culture Supernatants

A549, HCA-7 or HeLa cells (0.25 × 10^6^, 0.5 × 10^6^ and 0.15 × 10^6^ cells per well, respectively) were seeded in complete growth medium into six-well plates and allowed to attach for 24 h at 37 °C/5% CO_2_. The incubation was started by replacing medium with either 2 ml complete growth medium plus IL-1β 1 ng mL^−1^ and TNFα 5 ng mL^−1^(HeLa), complete growth medium containing only 2% FCS plus IL-1β 1 ngmL^−1^ (A549) or complete growth medium alone (HCA-7) plus the inhibitors. Cells were incubated for 4 h (HCA-7), 16 h (HeLa) or 24 h (A549). For ARA add-back experiments, HeLa cells were additionally incubated with different concentrations of ARA (5, 10 and 20 µM) in the presence of the inhibitors for 5 h. Afterwards, media were collected on ice and subsequently centrifuged (940 × *g*, 5 min, 4°C) to remove cell debris. Then, PGE_2_ concentrations in the cell free supernatants were analyzed *via* LC-MS/MS or ELISA method.

### Intracellular PGE_2_ and Arachidonic Acid Release

HeLa cells (2.4 × 10^6^ per sample) were suspended in 9.5 ml complete growth medium containing cytokines (1 ng mL^−1^ IL-1ß; 5 ng mL^−1^ TNFβ) and were seeded on Petri dishes (9.4 cm diameter, surface untreated). Cells were incubated at 37°C/5% CO_2_ for 60 (ARA) or 300 (PGE_2_) min. To stop the incubation, cell suspensions were transferred to falcon tubes and immediately chilled on ice. Subsequently, samples were centrifuged (340 g, 4 °C, 5 min) and the cell free supernatants were frozen at −80°C. The remaining cell pellets were washed twice with ice-cold PBS containing 1 mg mL^−1^ fatty acid-free BSA to eliminate extracellular lipids and then stored at -80 °C. Detection of intracellular PGs and ARA in the cell pellets was carried out *via* LC-MS/MS analysis.

### Human Whole-Blood Assay

Aliquots of heparinized human whole blood (500 µL) from healthy donors were incubated with LPS (10 μg mL^−1^) plus inhibitors or vehicle (DMSO) for 24 h at 37 °C. Platelet COX-1 activity was halted by addition of aspirin (100 µM). Plasma was then separated from cells by centrifugation at 1,000 × g, 4°C for 15 min and PGs in the plasma supernatants were evaluated by LC-MS/MS. Blood samples were drawn with the informed consent of the donors.

### Extraction and Detection of Prostaglandins and Arachidonic Acid *Via* LC-MS/MS

PGs were extracted from 200 µL of cell culture supernatants or cell pellets twice with 600 µL ethyl acetate after addition of the corresponding internal standards. The organic layer containing the analytes of interest was transferred to an autosampler amber glass vial and evaporated at 45°C under a gentle stream of nitrogen. For PG analysis, samples were reconstituted after extraction with 50 µL acetonitrile/water/formic acid (20:80:0.0025 v/v), centrifuged for 2 min at 10,000 × *g* and transferred to glass vials (Machery-Nagel, Düren, Germany) before they were injected into the LC-MS/MS system (45 µL). For chromatographic separation, a Synergi Hydro-RP column (150 mm × 2 mm I.D., 4 µm) and pre-column AQUA C18 were used (Phenomenex, Aschaffenburg, Germany). The mobile phase was gradually mixed from mobile phases A and B during the run with a flow rate of 0.3 ml min^−1^: mobile phase A water/formic acid (100:0.002 v/v); mobile phase B acetonitrile/formic acid (100:0.0025, v/v). Mass spectrometric detection was conducted with a hybrid triple quadrupole-ion trap tandem mass spectrometer, 5500 QTrap (AB SCIEX, Darmstadt, Germany) equipped with an electrospray ion source (ESI) operated in negative mode. Quantification of the analytes was performed employing Analyst^®^ Software (Version 1.5; AB Sciex, Darmstadt, Germany). For a detailed description of the method used to detect PGs, see (Sisignano et al., 2013).

### Detection of PGE_2_ *Via* Enzyme-Linked Immunosorbent Assay Technique

For detection of PGE_2_ *via* ELISA (Correlate-EIATM, Prostaglandin E_2_ Enzyme Immunoassay Kit, Assay Designs, Inc., Ann Arbor, MI, United States), supernatants and standards were diluted with complete growth medium. The ELISA was then carried out according to the manufacturer’s protocol using a microplate reader (Infinite M200, Tecan Group Ltd., Crailsheim, Germany).

### Protein Extraction and Western Blot Analysis

The cells were harvested in 4 ml ice-cold PBS and centrifuged at 1,000 × *g* for 5 min at 4°C. The cell pellet was then washed once with 1 ml ice-cold PBS. This was followed by resuspension in extraction buffer [PBS pH 7.4, 1% TritonX100, protease inhibitor cocktail tablets (Complete Mini; Roche Diagnostic GmbH, Mannheim, Germany)]. Subsequently, cells were subjected to freeze-thawing and the resulting cell homogenates were centrifuged at 10,000 × *g* for 10 min at 4°C. For detection of MRP-4 cells pellets were alternatively lysed in a buffer containing 10 mM Tris-HCl (pH 7.4), 20 mM Chaps and 0.5 mM EDTA plus protease inhibitor cocktail tablets. Protein concentrations in the supernatants were determined using the bicinchoninic acid (BCA) protein assay (Pierce, Thermo Fisher Scientific, Rockford, IL, United States). Equal protein quantities per sample were then separated by 10% sodium dodecyl sulfate-polyacrylamide gel electrophoresis (SDS-PAGE) and proteins were electrophoretically blotted onto nitrocellulose membranes (Hybond-C Extra, Amersham Biosciences Ltd., Little Chalfont, United Kingdom). Following drying, membranes were incubated overnight in Odyssey blocking reagent (LI-COR Biosciences, Bad Homburg, Germany). Membranes were then treated with the respective primary antibodies directed against COX-2, mPGES-1 (Cayman Chemical; Ann Arbor, MI, United States), cPLA_2α_ (C-20), MRP-4 (N-18) and ß-actin (I-19) (Santa Cruz Biotechnology, Heidelberg, Germany), ß-actin (Sigma-Aldrich, Munich, Germany) and 15-PG dehydroxygenase (15-PGDH; ab96732; Abcam, Cambridge, United Kingdom). All antibodies were diluted in Odyssey blocking reagent. Membranes were thoroughly rinsed with PBS containing 0.2% Tween 20, and were then incubated with an IRDye680- or IRDye800-conjugated secondary antibody (LI-COR Biosciences, Bad Homburg, Germany) in Odyssey blocking reagent. Protein-antibody complexes were visualized on the Odyssey Infrared Imaging System (LI-COR Biosciences, Bad Homburg, Germany).

### Activity Assay With Isolated COX-1 and COX-2 Isoenzymes

Recombinant human COX-2 and ovine COX-1 were assayed with the COX Inhibitor Screening Assay Kit from Cayman Chemical (Ann Arbor, MI, United States) according to the manufacturer’s instructions. The read-out of the ELISA reaction was carried out with a microplate reader (Infinite M200, Tecan Group Ltd., Crailsheim, Germany).

### MRP-4-Efflux Assay

Hek293/4.63 cells overexpressing MRP-4 were harvested and washed once with transport buffer (Hank’s balanced salt solution w/o Ca^2+^, Mg^2+^ supplemented with 0.01% BSA and 10 mM HEPES). 0.85 × 10^6^ cells per mL and sample were plated on 24-well plates and treated with inhibitors for 15 min at room temperature. Thereafter, 1 µM BCECF-AM was added and cells were further incubated at 37°C in 5% CO_2_ for 30 min. Subsequently, cells were harvested, centrifuged (940 × *g*, 4°C, 5 min) and washed twice with ice-cold incubation buffer. Resulting cell pellets were lysed in incubation buffer containing 1% TritonX-100 for 30 min at room temperature. Next, cell lysates were assayed for fluorescence activity (excitation λ: 505 nm, emission λ: 560 nm) utilizing a standard curve. Total protein content was assessed through the BCA method. The fluorescence and VIS readout was determined with a microplate reader (Infinite M200, Tecan Group Ltd., Crailsheim, Germany).

### Confocal Microscopy

HeLa cells were seeded onto glass cover slips in 12-well plates. The next day, the cells were incubated with 1 ng mL^−1^ IL-1β and 5 ng mL^−1^ TNFα in the presence of the 5-LO inhibitors for 30 min. After this, cells were washed twice with ice-cold PBS followed by fixation with 4% paraformaldehyde in PBS for 30 min. Subsequently, cells were washed and then blocked and permeabilized with PBS containing 1% BSA plus 0.2% Triton X-100 for 60 min at room temperature. Thereafter, the cells were incubated overnight with the cPLA_2α_ antibody (goat, C-20, Santa Cruz Biotechnology, Heidelberg, Germany) in antibody buffer (PBS + 0.1% BSA + 0.2% Triton X-100). The next day, the cells were washed again and incubated with a secondary anti-goat IgG antibody conjugated with Alexa Fluor 568 (Thermo Fisher Scientific, Waltham, United States) for 60 min. Nuclei were then stained with DAPI (1 μg mL^−1^, 5 min) and cover slips were mounted with mowiol onto microscope slides. Images were acquired by a Leica TCS-SP5 confocal microscope (Leica, Wetzlar, Germany) under identical conditions for pinhole opening, laser power, photomultiplier tension and layer number. During data elaboration by Fiji software (https://fiji.sc), identical parameters were applied for all samples.

### Statistical Analysis

All data are presented as mean + SD (standard deviation). GraphPad Prism version 5.00 (GraphPad Software, San Diego, CA, United States) was used for statistical analysis. Data were subjected to one-way analysis of variance (ANOVA) coupled with Dunnett’s post *t*-test for multiple comparisons. A sigmoidal concentration-response curve-fitting model with a variable slope was employed to calculate the IC_50_ values (non-linear regression; dose-response-inhibition; log(inhibitor) vs. response- variable slope (4 parameters)). Equation used for the fitting $Y=\frac{Bottom\;+\left(Top-Bottom\right)}{\left(1+10^{\left(\left(\log IC50-X\right)\times HillSlope\right)}\right)}$ .

## Results

### Influence of the 5-LO Inhibitors on PGE_2_ Release From HeLa, A549 and HCA-7 Cells

**FIGURE 2:**
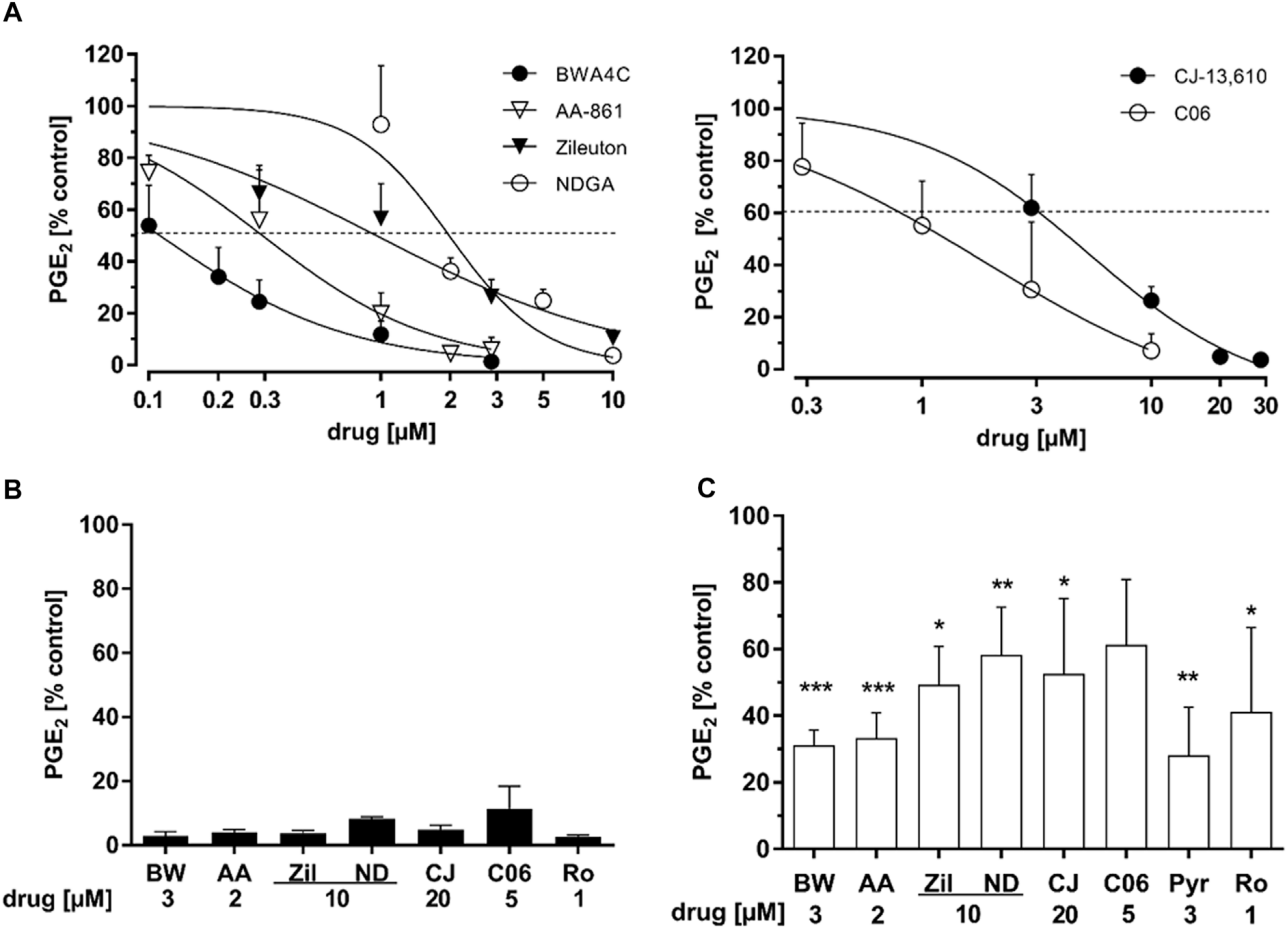
Inhibition of prostaglandin E_2_ levels in HeLa, A549 and HCA-7 cell supernatants. **(A)** HeLa cells were treated with cytokines (IL-1β 1 ng mL^−1^, TNFα 5 ng mL^−1^) plus increasing concentrations of the 5-LO inhibitors AA-861, BWA4C, NDGA, zileuton, CJ-13,610 or C06 for 16 h **(B)** A549 cells were treated with IL-1β (1 ng mL^−1^) and the 5-LO inhibitors for 24 h **(C)** HCA-7 cells were treated with the 5-LO inhibitors for 4 h. PG concentrations in the cells supernatants were assessed by LC-MS/MS. 1 µM rofecoxib and 3 μM Pyr were used as positive controls for COX-2 and cPLA_2α_ inhibition, respectively. For detailed methodical descriptions, see the Materials and Methods section. Mean PG release into HeLa cell supernatants: PGE_2_ 22.6 ± 11.99 ng mL^−1^; A549 cells: PGE_2_ 12.53 ± 2.04 ng mL^−1^; HCA-7 cells: PGE_2_ 4.69 ± 0.78 ng mL^−1^. Values represent the mean + SD of three independent experiments. AA, AA-861; BW, BWA4C; CJ, CJ-16,310; ND, NDGA; Pyr, cPLA_2α_ inhbitor; Ro, rofecoxib; Zil, zileuton.

HeLa cervix carcinoma cells do not express 5-LO and readily upregulate PGE_2_ formation upon treatment with TNFα and IL-1β (T/IL) within hours (Kahnt et al., 2013). This is driven by the induction of COX-2 and mPGES-1 expression as well as cPLA_2α_ mediated arachidonic acid liberation. To study the influence of 5-LO inhibitors of PG biosynthesis several frequently used 5-LO inhibitors with distinct modes of inhibition were chosen: AA-861, BWA4C, C06, CJ-13,610 and zileuton as well as the pan-LO inhibitor NDGA (Figure 1). HeLa cells were treated overnight (16 h) with 5 ng mL^−1^ TNFα and 1 ng mL^−1^ IL-1β plus the inhibitors and PGE_2_ concentrations in the cell culture supernatants were analyzed via LC-MS/MS. All 5-LO inhibitors dose-dependently blocked PGE_2_ levels in HeLa cell supernatants (Figure 2A) with IC_50_ values in the low micromolar range. As can be seen in Table 1 the inhibitory potency for PGE_2_ in HeLa cell supernatants was comparable to the 5-LO inhibitory potencies of the inhibitors reported in the literature. To rule out cell line specific effects, inhibition of PGE_2_ was also assessed in the supernatants of IL-1β (1 ng mL^−1^, 24 h) treated A549 lung carcinoma cells as well as HCA-7 colon carcinoma cells showing constitutive PGE_2_ formation (4 h). In A459 cells, all 5-LO inhibitors attenuated PGE_2_ accumulation into the cell supernatants (Figure 2B) with comparable efficacy to HeLa cells. In HCA-7 cells, PGE_2_ levels were also inhibited, albeit with lower efficacy compared to HeLa and A549 cells (Figure 2C). Interestingly, PGE_2_ inhibition by the cPLA_2α_ inhibitor Pyr and the selective COX-2 inhibitor rofecoxib was weak in HCA-7 cells as well.

**TABLE 1:** Comparison of literature-derived IC_50_ values for 5-LO inhibition and IC_50_ values obtained for inhibition of PG release in cytokine stimulated HeLa cells and human whole blood preparations. IC_50_ values for inhibition of 5-LO activity were obtained from the literature (Ménard et al., 1990; Fischer et al., 2010; Hofmann et al., 2012). CI, confidence interval; NDGA, nordihydroguaiaretic acid; SD, standard deviation; WB, human whole blood.

[µM]		AA-861	BWA4C	C06	CJ13,610	NDGA	zileuton
5-LO		**0.01**	**0.01**	**0.7**	**0.2**	**0.6**	**0.3**
PGE_2_	**Mean** **± SD**	**0.4 ± 0.2**	**0.1 ± 0.1**	**1.2 ± 0.4**	**4.4 ± 1.4**	**1.4 ± 0.1**	**1 ± 0.3**
HeLa^a^	**CI**	≤ 0.8^c^	≤ 0.2^c^	0.3–2	0.9 –– 7.8	1–1.7	0.3–1.6
PGE_2_	**Mean** **± SD**	**31.1 ± 12.6**	**3.7 ± 1.9**	**> 10**	**8.4 ± 2.4**	**> 100**	**26.9 ± 6.4**
WB^b^	**CI**	≤ 62.5^c^	≤ 8.6^c^	-	11 – 42.8	-	2.4–14.3
TXB_2_	**Mean** **± SD**	**33.8 ± 5.1**	**7.6 ± 3.5**	**> 10**	**39.5 ± 8.8**	**> 100**	**40.8 ± 19.2**
WB^c^	**CI**	31.1–56.5	≤ 16.4^c^	-	≤ 88.6^c^	-	17.6–61.4

a Cell supernatants of cytokine-treated HeLa cells (16 h).

b LPS- stimulated human whole blood (24 h).

c Lower limit of the confidence interval not quoted as below zero.

### Influence of the 5-LO Inhibitors on Serum Prostaglandin Levels in LPS-Stimulated Human Whole Blood

**FIGURE 3:**
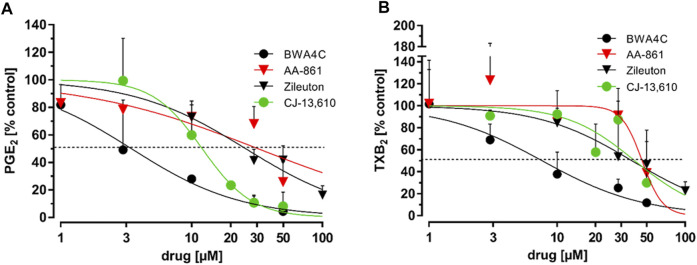
Inhibition of PGE_2_ and TXB_2_ in LPS-stimulated human whole blood. Freshly drawn human whole blood was stimulated with bacterial LPS (10 μg mL^−1^, 24 h). Simultaneously, the blood was treated with increasing concentrations of AA-861, BWA4C, zileuton or CJ-13,610. Plasma PG concentrations were assessed by LC-MS/MS. For detailed methodical descriptions, see the Materials and Methods section. Mean PG formation in human whole blood: PGE_2_ 34.36 ± 8.29 ng mL^−1^, TXB_2_ 15.89 ± 6.60 ng mL^−1^; Values represent the mean + SD of three independent experiments.

LPS stimulated human whole blood serves as a model to predict the *in vivo* efficacy of compounds on PG synthesis (Patrignani et al., 1994). We therefore treated freshly drawn human whole blood, in which COX-1 activity was halted by aspirin (100 µM), with bacterial LPS (10 μg mL^−1^) for 24 h. Simultaneously, the 5-LO inhibitors or a vehicle control (DMSO) were added. 1 µM rofecoxib served as positive control. After this, sera were collected and the PG concentrations were analyzed using LC-MS/MS technique as described before (Kahnt et al., 2013). The 5-LO inhibitors AA-861, BWA4C, CJ-13,610 and zileuton blocked the accumulation of PGE_2_ and TXB_2_ in serum in a concentration-dependent manner (Figure 3). For IC_50_ values see Table 1. Of note, C06 in a concentration up to 10 µM and NDGA in a concentration up to 100 µM failed to suppress 5-LO activity by more than 50% in whole blood preparations.

### Effect of the 5-LO Inhibitors on Expression of Enzymes Involved in PGE_2_ Biosynthesis

A number of non-steroidal anti-inflammatory drugs (NSAIDs) are known to disrupt pro-inflammatory cytokine signaling by inhibition of transcription factors such as NFκB and AP-1 (Tegeder et al., 2001) in addition to their COX inhibitory properties. Therefore, we investigated whether the 5-LO inhibitors used in this study influence the expression of enzymes involved in PGE_2_ biosynthesis in cytokine-stimulated HeLa cells. The cells were treated with T/IL in the presence of the 5-LO inhibitors for 16 h and protein expression of cPLA_2α_, COX-2 and mPGES-1 was evaluated by Western blotting. The glucocorticoid dexamethasone served as positive control. Expression of cPLA_2α_ which releases ARA from intracellular membranes for eicosanoid formation did not change during T/IL treatment and neither the 5-LO inhibitors nor dexamethasone had an influence here (Supplementary Figure S1A). Next, expression of COX-2, the central enzyme in PG biosynthesis catalyzing the formation of the PG precursor PGH_2_, was determined. COX-2 was strongly upregulated by T/IL treatment and dexamethasone potently inhibited this upregulation. However, all 5-LO inhibitors tested failed to significantly interfere with COX-2 expression levels (Supplementary Figure S1B). Expression of microsomal PGE_2_ synthase-1 (mPGES-1), the COX-2 downstream synthase for PGE_2_ formation, was only moderately upregulated by T/IL treatment and dexamethasone as well as the 5-LO inhibitors failed to attenuate its expression in a significant manner (Supplementary Figure S1C).

### Impact of Exogenous Arachidonic Acid on 5-LO Inhibitor-Mediated PGE_2_ Inhibition

**FIGURE 4:**
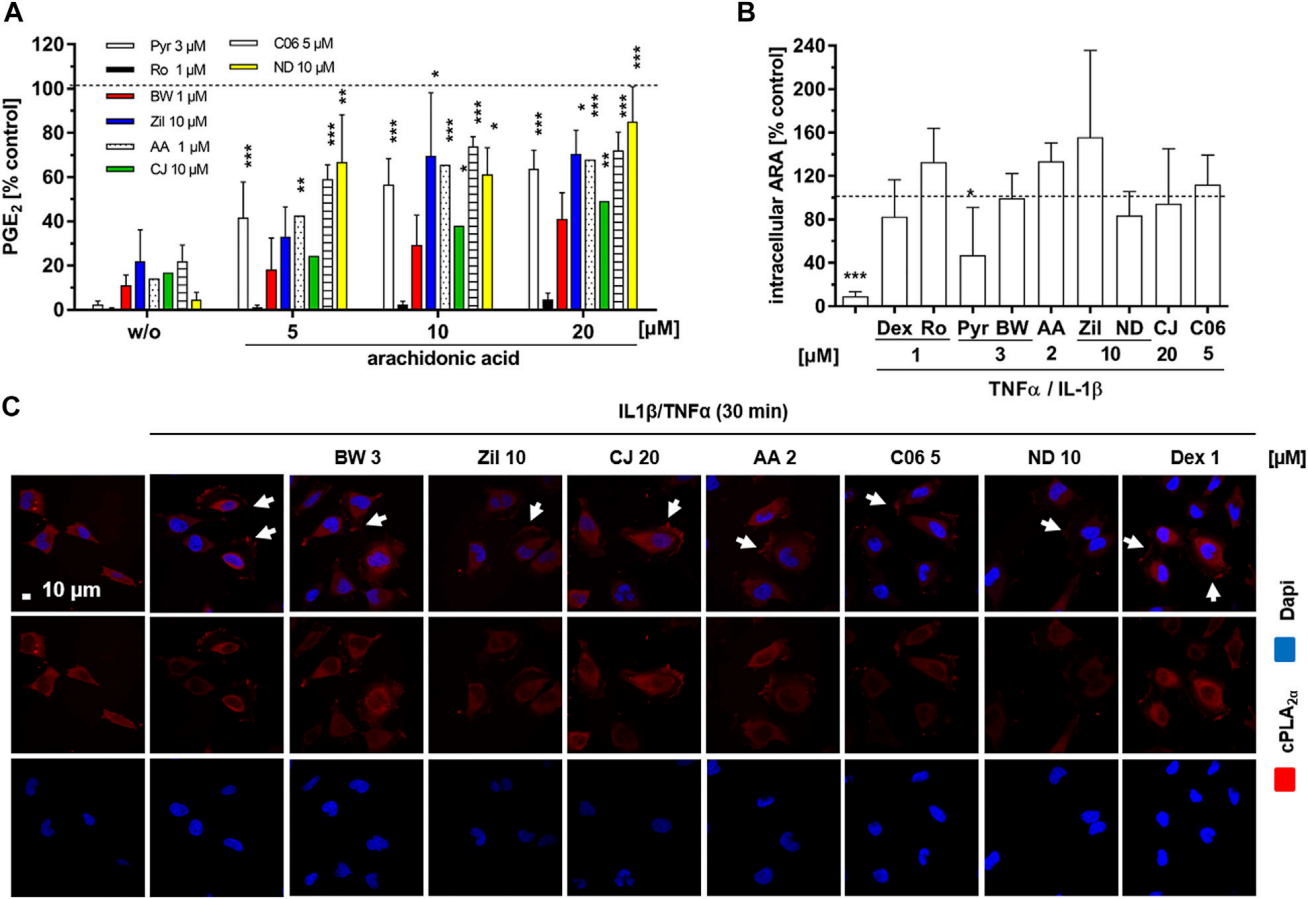
Influence on cPLA_2α_ translocation and arachidonic acid liberation in HeLa cells. **(A)** HeLa cells were treated with cytokines (IL-1β 1 ng mL^−1^, TNFα 5 ng mL^−1^), the inhibitors (1 μM AA-861, 10 µM zileuton, 10 µM CJ-13,610, 3 μM BWA4C, 10 µM NDGA, 5 µM C06) or vehicle (DMSO) plus increasing concentrations of ARA (5–20 µM) for 5 h and PGE_2_ concentrations in the cell culture supernatants were assessed by ELISA. The COX-2 inhibitor rofecoxib (1 µM) and the cPLA_2α_ inhibitor Pyr (3 µM) were used as controls. Mean PGE_2_ release from cytokine treated (5 h) HeLa cells: without ARA 4.69 ± 1.3 ng mL^−1^; with 5 µM ARA 29.09 ± 5.2 ng mL^−1^; with 10 µM ARA 31.6 ± 4.87 ng mL^−1^; with 20 µM ARA 40.16 ± 6.97 ng mL^−1^. **(B)** To measure intracellular ARA liberation, HeLa cells were treated with cytokines (IL-1β 1 ng mL^−1^, TNFα 5 ng mL^−1^) and the inhibitors (2 μM AA-861, 10 µM zileuton, 20 µM CJ-13,610, 3 μM BWA4C, 10 µM NDGA, 5 µM C06) or vehicle (DMSO) for 1 h and intracellular ARA was assessed by LC-MS/MS. 1 µM dexamethasone, 1 µM rofecoxib and 3 μM Pyr served as controls. Mean intracellular ARA concentration in cytokine treated (1 h) HeLa cells was 12.47 ± 4.69 ng Mio^−1^ cells. **(C)** Confocal microscopy to investigate cPLA_2α_ translocation in HeLa cells treated with cytokines (IL-1β 1 ng mL^−1^, TNFα 5 ng mL^−1^) and the inhibitors (2 μM AA-861, 10 µM zileuton, 20 µM CJ-13,610, 3 μM BWA4C, 10 µM NDGA, 5 μM C06, 1 µM dexamethasone) or vehicle control (DMSO) for 30 min. Values represent mean + SD of three independent experiments. AA, AA-861; BW, BWA4C; CJ, CJ-16,310; Dex, dexamethasone; ND, NDGA; Pyr, cPLA_2α_ inhibitor; Ro, rofecoxib; Zil, zileuton. *, *p* ≤ 0.05; **, *p* ≤ 0.01; ***, *p* ≤ 0.001.

As enzyme expression levels of cPLA_2α_, COX-2 and mPGES-1 were not significantly affected by the 5-LO inhibitors, we excluded interference with the transcription/translation machinery and downstream signal transduction cascades as the underlying mechanism for PGE_2_ inhibition. Thus, we next focused on enzyme activities along the PG synthesis cascade. For this, we studied the effects of exogenous addition of ARA to cytokine (T/IL)-treated HeLa cells. The cells were supplemented with different concentrations of ARA (5–20 µM) for 5 h in the presence of 5-LO-inhibitors and PGE_2_ concentrations in the cell supernatants were assessed and compared to ARA untreated cells. Rofecoxib (1 µM) and Pyr (3 µM) served as positive controls for COX-2 and cPLA_2α_ inhibition, respectively. PGE_2_ release from HeLa cells was elevated by ARA supplementation in a concentration-dependent manner (Figure 4A). The control compounds rofecoxib and Pyr inhibited PGE_2_ levels to a comparable extent in cells without exogenous ARA supplementation but increasing exogenous ARA levels reversed the inhibitory potency of Pyr from over 95–∼ 30% inhibition, probably by circumventing intracellular ARA release while rofecoxib mediated PGE_2_ inhibition was not influenced. Comparable to Pyr, PGE_2_ release in all samples treated with the 5-LO inhibitors was restored with rising ARA concentrations. This was a compound-specific effect ranging from 40% remaining PGE_2_ levels in BWA4C to 90% for NDGA treated cells at an ARA concentration of 20 µM.

### Influence of the 5-LO Inhibitors on Arachidonic Acid Liberation

ARA liberation from membrane phospholipids by cPLA_2α_ is one of the rate-limiting steps in PG biosynthesis. In its inactive form the enzyme resides in the cytosol. Upon cytokine stimulation, the enzyme is phosphorylated and translocates to perinuclear membrane compartments where it facilitates the release of ARA from membrane phospholipids within minutes. Since all 5-LO inhibitors tested in this study displayed reversible PGE_2_ inhibition in the presence of increasing ARA concentrations, their effect on inhibition of cPLA_2α_ translocation and intracellular ARA release was further investigated in HeLa cells. Cytokine (T/IL) treatment elevated free intracellular ARA by approximately 10-fold compared to the untreated control after 60 min. The cPLA_2α_ inhibitor control Pyr (3 µM) reversed this effect by 50% (Figure 4B). Dexamethasone (1 µM) as well as the 5-LO inhibitors did not affect T/IL-induced ARA liberation.

**FIGURE 5:**
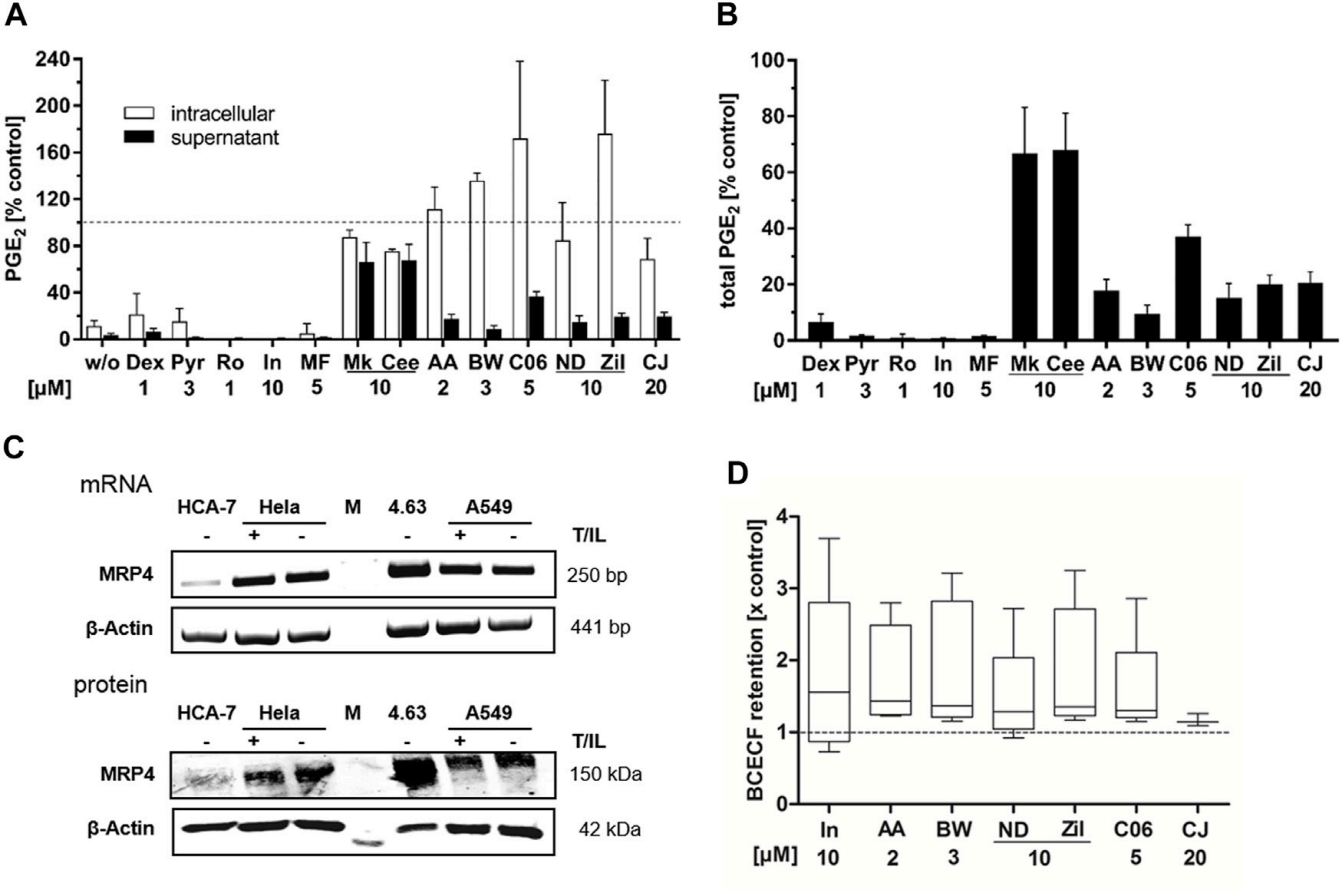
Influence on intracellular PGE_2_ levels and prostanoid transport. HeLa cells were treated with cytokines (IL-1β 1 ng mL^−1^, TNFα 5 ng mL^−1^) and the inhibitors (2 μM AA-861, 10 µM zileuton, 20 µM CJ-13,610, 3 μM BWA4C, 10 µM NDGA, 5 µM C06) or vehicle (DMSO) for 5 h. Unstimulated cells served as negative control (w/o). Afterwards, intracellular and extracellular PGE_2_ concentrations were assessed separately by LC-MS/MS. The glucocorticoid dexamethasone (1 µM), the COX-2 inhibitor rofecoxib (1 µM), the COX-1/-2/MRP-4 inhibitor indomethacin (10 µM), the cPLA_2α_ inhibitor Pyr (3 µM), the mPGES-1 inhibitor MF-63 (5 µM) and the MRP-4 inhibitors Mk-571 and Ceefourin-1 (10 µM each) were used as controls. **(A)** Intracellular and extracellular PGE_2_ levels relative to the vehicle control and **(B)** total PGE_2_ formed relative to the vehicle control. Mean intracellular PGE_2_ formation was 0.82 ± 0.50 ng•mio^−1^ cells and extracellular 18.24 ± 4.74 ng mL^−1^. Values represent mean + SD of three independent experiments. **(C)** mRNA and protein expression of MRP-4 in HeLa, A549, HCA-7 and Hek293/4.63 cells. **(D)** Influence of the inhibitors (2 μM AA-861, 10 µM zileuton, 20 µM CJ-13,610, 3 μM BWA4C, 10 µM NDGA, 5 µM C06) on BCECF export from MRP-4-overexpressing Hek293/4.63 cells. Indomethacin was used as positive control. Values are presented as box and whisker plots (Tukey style) and represent data from three to five independent experiments. AA, AA-861; BW, BWA4C; CJ, CJ-16,310; Dex, dexamethasone; In, indomethacin; M, marker; MF, MF-63; ND, NDGA; Pyr, cPLA_2α_ inhibitor; Ro, rofecoxib; T/IL, IL-1β/TNFα; Zil, zileuton.

Next, intracellular cPLA_2α_ translocation was assessed via confocal microscopy in HeLa cells treated with cytokines (T/IL) for 30 min. Upon cytokine treatment, HeLa cells changed cell morphology and showed enhanced spreading on the two-dimensional surface. cPLA_2α_ was evenly distributed in the cytosol and translocated to a perinuclear compartment as well as to foci in the cytoplasmic membrane upon stimulation (Figure 5B, also published by (Schievella et al., 1995; Grewal et al., 2003; Grewal et al., 2005)). This cytokine-induced cellular redistribution of cPLA_2α_ was not altered by any of the tested 5-LO inhibitors (Figure 4C). Interestingly, the glucocorticoid dexamethasone also failed to interfere with translocation of cPLA_2α_.

### Influence of the 5-LO Inhibitors on the Activity of Purified COX Isoenzymes

Since the 5-LO inhibitors had no effect on intracellular ARA release and cPLA_2α_ distribution, cyclooxygenases (COX-1 and COX-2) were next considered to be the target responsible for PGE_2_ inhibition in HeLa cell supernatants. Since T/IL stimulated HeLa cells express both COX isoenzymes, we conducted activity assays with purified COX-1 and -2 enzymes. For this, recombinant human COX-2 or ovine COX-1 were pre-treated with the 5-LO inhibitors. Subsequently, the reaction was started by addition of ARA. Afterwards, formed PGH_2_ was reduced to PGF_2α_ by stannous chloride and analyzed via ELISA technique. 1 µM rofecoxib (1 µM) and 1 µM sc-560 were employed as positive controls for COX-2 and COX-1, respectively and the DMSO control was set to 100%. As expected, rofecoxib and sc-560 potently inhibited COX-2 and COX-1 enzyme activity, respectively. In contrast, samples treated with the 5-LO inhibitors in concentrations that completely blocked PGE_2_ in HeLa supernatants, did not show any inhibition of both COX isoenzymes (Supplementary Figures S2A, S2B).

### Effect of the 5-LO Inhibitors on PGE_2_ Metabolism and Transport

ARA release as well as PGH_2_ and PGE_2_ biosynthesis were not directly impaired by the 5-LO inhibitors. Next, the expression of 15-hydroxyprostaglandin dehydrogenase (15-PGDH), the enzyme responsible for metabolic PGE_2_ inactivation, was investigated. For this, HeLa cells were treated with cytokines (T/IL) in the presence of the 5-LO inhibitors for 16 h and 15-PGDH expression was determined. All inhibitors tested did not influence the expression of this enzyme (Supplementary Figure S2C). We also sought to measure intracellular formation of 15-ketoPGE_2_, the PGE_2_ metabolite formed by 15-PGDH, *via* LC-MS/MS technique. This metabolite could not be detected in our HeLa cell preparations and was therefore not produced in high amounts.

Next, PGE_2_ export from T/IL treated HeLa cells was investigated. The cells were incubated with the cytokines and inhibitors for 5 h followed by analysis of intra- and extracellular PGE_2_ levels. For this, cell supernatants as well as intact cells from the same sample were extracted separately and PGE_2_ concentrations were measured via LC-MS/MS. As expected, the control compounds dexamethasone, rofecoxib (COX-2 inhibitor), Pyr (cPLA_2α_ inhibitor), indomethacin (COX-1/-2 inhibitor) and MF-63 (mPGES-1 inhibitor) equipotently inhibited extra- and intracellular PGE_2_ concentrations (Figure 5A). The MRP-4 inhibitors Mk-571 and Ceefourin-1 inhibited the extracellular PGE_2_ levels by about 35% but did not lead to intracellular PGE_2_ accumulation. In contrast, the 5-LO inhibitors strongly attenuated extracellular while elevating intracellular PGE_2_. From the comparison of the total PGE_2_ formed in the incubations (intracellular + extracellular) it can be seen that all 5-LO inhibitors attenuated the total PGE_2_ levels by 63 up to 90% depending on the compound. Compounds directly interfering with components of the PGE_2_ biosynthesis machinery such as dexamethasone, Pyr, rofecoxib, indomethacin and MF63 inhibited total PGE_2_ by 95–99%.

Since inhibition of the prostaglandin exporter MRP-4 by Mk-571 and Ceefourin-1 partly reduced the PGE_2_ release from HeLa cells, we investigated if inhibition of this transporter is part of the 5-LO inhibitor mediated attenuation of extracellular PGE_2_. First, we confirmed that all cell lines used in this study express MRP-4. For this, we isolated total mRNA and protein from cytokine-treated HeLa and A549 cells as well as HCA-7 cells. HEK293/4.63 cells overexpressing MRP-4 were used as positive control (Reid et al., 2003). Indeed, all cell lines investigated in this study expressed MRP-4 on the mRNA and protein level (Figure 5C). Then, the effect of the 5-LO inhibitors on MRP-4 efflux activity was investigated. For this, the export of a fluorescent dye (2',7'-bis-(2-carboxyethyl)-5-(and-6)-carboxyfluorescein; BCECF) from MRP-4 overexpressing HEK293/4.63 was measured. In parallel a P-glycoprotein (P-gp) antagonist (1 µM PSC833) was added to block BCECF transport via P-gp. 10 µM indomethacin were used as positive control. All inhibitors tested induced a more or less pronounced retention of BCECF compared to the DMSO treated control (Figure 5D).

## Discussion

It is widely accepted that 5-LO-derived LTs contribute to a large number of chronic inflammatory diseases. While their involvement in atopic disorders is well documented, their role in rheumatoid arthritis, atherosclerosis and cancer still remains somewhat elusive. A large number of preclinical studies on the role of 5-LO in neurological and cardiovascular diseases as well as cancer is based on pharmacological inhibition of the enzyme. Here, 5-LO inhibitors were found to efficiently ameliorate disease severity. According to this, the beneficial effects seen with these compounds are attributed to 5-LO inhibition and the reduction in LT formation although the compounds display great heterogeneity concerning their efficacy. This suggests additional off-target effects (Werz and Steinhilber, 2006; Steinhilber et al., 2010; Steinhilber, 2016).

LTs and PGs share the precursor fatty acid ARA and a number of drugs modifying LT or PG biosynthesis and signaling have already been reported to interact with targets in each of the other pathways. This has recently been shown for the 5-LO activating protein inhibitor MK-886 as well as the marketed CysLT1 receptor antagonists. These compounds exert additional mPGES-1 inhibition (Mancini et al., 2001; Kahnt et al., 2013). In addition, the cyclooxygenase inhibitors celecoxib and sulindac sulfide were found to inhibit 5-LO and the 5-LO inhibitor zileuton has been shown to interfere with intracellular ARA liberation (Maier et al., 2008; Rossi et al., 2010; Steinbrink et al., 2010; Kahnt et al., 2013).

In this study, we decided to investigate the PG inhibitory potential of five frequently used 5-LO inhibitors (AA-861, BWA4C, CJ-13,610, zileuton, C06) as well as the pan-LO inhibitor NDGA. The FLAP inhibitor MK-886 was not included due to its already known inhibitory effects on mPGES-1 (Mancini et al., 2001). Rev-5901 a direct 5-LO inhibitor was excluded from the experiments due to its high cytotoxicity in cell culture (Fischer et al., 2010). All inhibitors tested interfered with PGE_2_ release in different cell culture models such as cytokine-stimulated HeLa and A549 cells as well as HCA-7 cells displaying constitutive PGE_2_ release. The IC_50_ values obtained were close to the 5-LO inhibitory potential of the compounds (see Table 1). Thus, it can be expected that the use of these inhibitors in concentrations needed to sufficiently block LT formation in cell culture also strongly affect PG transport in parallel. Furthermore, AA-861, BWA4C, CJ-13,610 and zileuton dose-dependently decreased PG release in human whole blood preparations stimulated with bacterial lipopolysaccharide. Depending on the compound, potencies were 2–60 fold lower compared to the IC_50_ values obtained with Hela cells which was probably due to plasma protein binding effects. The effectivity in whole blood raises the possibility that in addition to inhibition of LT formation, extracellular PG levels are disturbed by these compounds in animals and humans. Indeed, zileuton peak plasma levels at 2,400 mg per day can reach a concentration of about 20 µM in humans which is close to the IC_50_ value obtained in our whole blood experiments. This fits to observations from clinical studies were 400 and 600 mg zileuton four times a day resulted in comparable immediate bronchodilatory responses but displayed marked dose-response effects after 1–2 weeks of dosing (Liu et al., 1996). Indeed, COX products such as TXA_2_, PGD_2_ and PGF_2α_ are increased in allergic asthma and trigger bronchconstriction, vasodilation, inflammation and fibrosis (Claar et al., 2015). In addition, PGs are known to influence immune cell maturation and differentiation (Harizi and Gualde, 2004; Sakata et al., 2010). Therefore, the additional anti-inflammatory effects seen under long-term zileuton therapy might be due to its additional interference with PG release.

To characterize the PGE_2_ inhibitory effects of the 5-LO inhibitors, HeLa cells which do not express 5-LO (Hoshiko et al., 1990) were chosen to rule out lipid mediator shunting of ARA into PG pathways as well as other effects of leukotriene signaling. Our study showed that the 5-LO inhibitors had no influence on expression of several enzymes involved in PGE_2_ biosynthesis and metabolism such as cPLA_2α_, COX-2, mPGES-1 and 15-PGDH. Furthermore, the inhibitors did not influence ARA liberation and PGH_2_ formation by both COX isoenzymes. mPGES-1 activity was ruled out due to the fact that all PGs measured in human whole blood were equipotently inhibited by the 5-LO inhibitors. Furthermore, intracellular PGE_2_ levels were not affected by the 5-LO inhibitors while the mPGES-1 inhibitor MF63 potently attenuated intracellular PGE_2_. This showed that neither expression nor activities of enzymes involved in PG biosynthesis were inhibited by the compounds. Finally, we found that the 5-LO inhibitors AA861, BWA4C, C06 and zileuton triggered intracellular PGE_2_ accumulation suggesting interference with PG transport. Treatment with NDGA and CJ-13,610 did not result in PGE_2_ accumulation in fact intracellular PGE_2_ levels were moderately inhibited by 15 and 30%, respectively but still inhibition of extracellular PGE_2_ was more potent (85 and 80%, respectively). This suggests that in addition to PGE_2_ transport, also PGE_2_ metabolism might be affected by these compounds.

The process of prostaglandin secretion is still not completely understood. Passive membrane diffusion of these lipid mediators is unlikely, due to their negative charge in aqueous solution under physiological pH as well as the presence of several hydroxyl groups in these molecules. This membrane impermeability has been shown in the past and strongly argues for active transport mechanisms to be involved in PG release (Bito and Baroody, 1975; Baroody and Bito, 1981; Chan et al., 1998). It has recently been shown that MRP-4 is a PG transporter and contributes to PGE_2_ release in HeLa cells (Reid et al., 2003). We confirmed expression of this transporter in all cell lines used in this study. Therefore, MRP-4 transport experiments were carried out in a MRP-4 over-expressing cell line (HEK293/4.63) in presence of the 5-LO inhibitors. Although, the effect was not very pronounced, all 5-LO inhibitors led to an accumulation of the fluorescent MRP-4 substrate BCECF. We also found that the MRP-4 inhibitors MK-571 and Ceefourin-1 reduced PGE_2_ in the HeLa cell supernatants by about 35%. This suggests that in addition to MRP-4, other transport mechanisms are also involved in PGE_2_ release in HeLa cells which are influenced by the 5-LO inhibitors. Preliminary data from our lab show that PGE_2_ can be stored and rapidly secreted from HeLa cells suggesting that exocytosis pathways are involved. In line with this, a recent study showed that the PG importer PGT (SLCO2A1) is involved in the loading of exogenous PGE_2_ into intracellular compartments in murine macrophages and human cancer cells resulting in elevated secretion of PGE_2_ via exocytosis (Shimada et al., 2015; Kasai et al., 2016). It has to be investigated in the future if this importer is also controlling PGE_2_ release in HeLa, A549 and HCA-7 cells and primary human leukocytes and if the 5-LO inhibitors interfere with PG exocytosis. Indeed, NDGA has been shown to induce protein redistribution between Golgi and ER by interference with the protein transport (Fujiwara et al., 1998) and is also able to disrupt the actin skeleton (Holland et al., 2001). Since PG transporters such as MRP-4 and PGT can shuttle between intracellular structures and the plasma membrane (Hoque and Cole, 2008; Schaletzki et al., 2017), the inhibitors might also disturb the intracellular distribution of the transporters and PG loaded vesicles leading to PG accumulation inside the cells.

When we compared the total PGE_2_ formation in HeLa cells calculated by addition of intracellular and extracellular PGE_2_, we found a 63–90% attenuation of total PGE_2_ by the 5-LO inhibitors. In comparison, inhibitors directly interfering with PGE_2_ biosynthesis via attenuation of enzyme expression or activity such as dexamethasone and rofecoxib inhibited total PGE_2_ between 95 and 99%. Apparently, potent inhibition of PGE_2_ release traps PGE_2_ formed in intracellular storage compartments. When these compartments reach their maximum an intracellular negative feedback loop seems to be operating that restricts further PG biosynthesis to prevent an overload of these compartments. Of note, ARA release is not attenuated here since our inhibitors had no influence on this (Figure 4B).

All 5-LO inhibitors tested showed a much lower PGE_2_ inhibitory potency in HCA-7 cells compared to HeLa and A549 cells as well as human whole blood. This loss of PGE_2_ inhibitory potency also applied to the COX-2 inhibitor rofecoxib and the cPLA_2α_ inhibitor Pyr in our study and has also been reported earlier for dimethylcelecoxib in these cells (Wobst et al., 2008). HCA-7 cells display stimulus-independent PGE_2_ formation. This suggests that an alternative route of PG secretion is existent in these cells. In addition, we found that inhibition of PG transport by the 5-LO inhibitors is of a competitive quality in HeLa cells as exogenous ARA counteracted the inhibitor-induced intracellular PGE_2_ accumulation. It is therefore conceivable that due to constant high intracellular PGH_2_/PGE_2_ levels in HCA-7 cells, competitive transport inhibitors might lose potency.

Evidence from literature supports the findings on the PG suppressive nature of the 5-LO inhibitors. NDGA was already shown to suppress ovulation in rat ovaries and yellow perch follicles inhibiting PGE_2_ and PGF_2α_ at the same time and AA-861 was found to suppress PGE_2_ formation in activated rat peritoneal macrophages (Ohuchi et al., 1983; Bradley and Goetz, 1994; Mikuni et al., 1998). These studies did not elucidate the underlying mechanisms and it was not clarified if the suppression of PG formation was a consequence of the inhibition of LT biosynthesis or an off-target effect of the compounds themselves. In line with our data, Rossi et colleagues recently published the inhibitory effect of zileuton on PGE_2_ formation (Rossi et al., 2010). The authors observed inhibition of ARA release from [3H]-labelled, LPS-stimulated murine J774 macrophages. From these data as well as translocation experiments and animal studies it was concluded that the cPLA_2α_-mediated release of ARA is the most likely target of zileuton action. In contrast to this, we here identified the interference with PG transport as the mechanism responsible for the 5-LO inhibitor-mediated PGE_2_ reduction. Rossi et al. did not investigate intracellular lipid mediator levels. Furthermore, the decrease in ARA-derived [3H]-labelled lipid mediators into the cell supernatant they found in J774 and peritoneal macrophages might have also derived from inhibition of eicosanoid export. This was not investigated further by the authors.

It is remarkable that although the scaffolds of the 5-LO inhibitors tested in this study are quite different, all compounds displayed the ability to suppress PG transport. All compounds tested in this study are thought to enter the catalytic center of 5-LO which suggests a certain similarity to the substrate ARA. LO and COX enzymes share ARA as substrate for conversion into LTs and PGs, respectively. It is therefore not surprising that many eicosanoid synthases, receptors and transporters display certain promiscuity. Consequently, eicosanoid mimetics targeting one enzyme/receptor involved in eicosanoid biosynthesis and signaling are likely to interfere with other eicosanoid pathway enzymes. This substrate-dependent cross-reactivity has already been observed for a number of non-steroidal anti-inflammatory drugs for transporters such as MRP-4 (Reid et al., 2003).

Several conclusions with relevance to future research on 5-LO can be drawn from the present study: (I) The pharmacological effects of 5-LO inhibitors are partly compound-specific and involve 5-LO-independent mechanisms. (II) Results from cell culture experiments on the role of 5-LO in inflammation or pain may be misleading when pharmacological inhibitors were used even at concentrations below < 1 µM. Thus, such inhibitor-related findings need to be confirmed by an independent methodology such as knock-down of 5-LO and add-back experiments making use of 5-LO products. (III) For the development of future 5-LO inhibitors, investigations on PG biosynthesis using a cell-based model in addition to studies using purified mPGES-1 and COX isoforms are important.

## Data Availability

The original contributions presented in the study are included in the article/Supplementary Material, further inquiries can be directed to the corresponding author.

## Abbreviations

ARA, arachidonic acid; FAF-BSA, fatty acid free bovine serum albumin; LC-MS/MS, liquid chromatography coupled to tandem mass spectrometry; LO, lipoxygenase; LT, leukotriene; PG, prostaglandin; T/IL, cytokine mix (IL-1β 1 ng mL^−1^ + TNFα 5 ng mL^−1^).
