# Supplementary material for: Inhibitors of Human 5-Lipoxygenase Potently Interfere With Prostaglandin Transport
Source: Front Pharmacol. 2022 Jan 21;12:782584. doi: 10.3389/fphar.2021.782584 (PMC8814463; doi:10.3389/fphar.2021.782584)
Supplement: Supplementary file 1 [file Presentation1.PPT]

## Slide 1
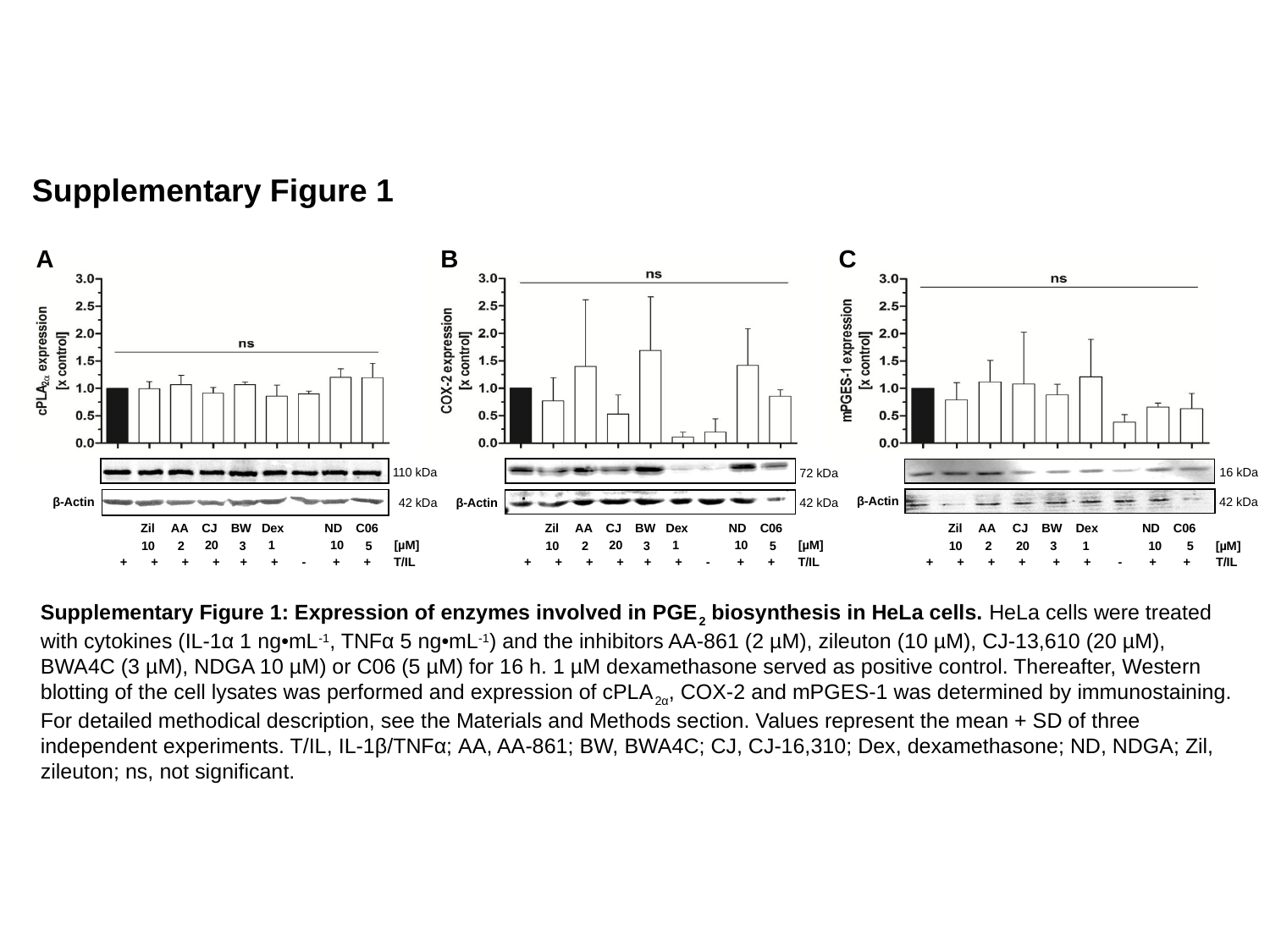

Supplementary Figure 1
C
B
A
 72 kDa
 42 kDa
 110 kDa
 β-Actin
 42 kDa
 16 kDa
 42 kDa
 β-Actin
 β-Actin
 AA CJ BW Dex ND C06
 Zil
[µM]
20
1
 10
10
 5
2
 3
T/IL
+ + + + + + - + +
 AA CJ BW Dex ND C06
 Zil
[µM]
20
1
 10
10
 5
2
 3
T/IL
+ + + + + + - + +
 AA CJ BW Dex ND C06
 Zil
 20
 1
 10
10
 5
2
 3
[µM]
T/IL
+ + + + + + - + +
Supplementary Figure 1: Expression of enzymes involved in PGE2 biosynthesis in HeLa cells. HeLa cells were treated with cytokines (IL-1α 1 ng•mL-1, TNFα 5 ng•mL-1) and the inhibitors AA-861 (2 µM), zileuton (10 µM), CJ-13,610 (20 µM), BWA4C (3 µM), NDGA 10 µM) or C06 (5 µM) for 16 h. 1 µM dexamethasone served as positive control. Thereafter, Western blotting of the cell lysates was performed and expression of cPLA2α, COX-2 and mPGES-1 was determined by immunostaining. For detailed methodical description, see the Materials and Methods section. Values represent the mean + SD of three independent experiments. T/IL, IL-1β/TNFα; AA, AA-861; BW, BWA4C; CJ, CJ-16,310; Dex, dexamethasone; ND, NDGA; Zil, zileuton; ns, not significant.

## Slide 2
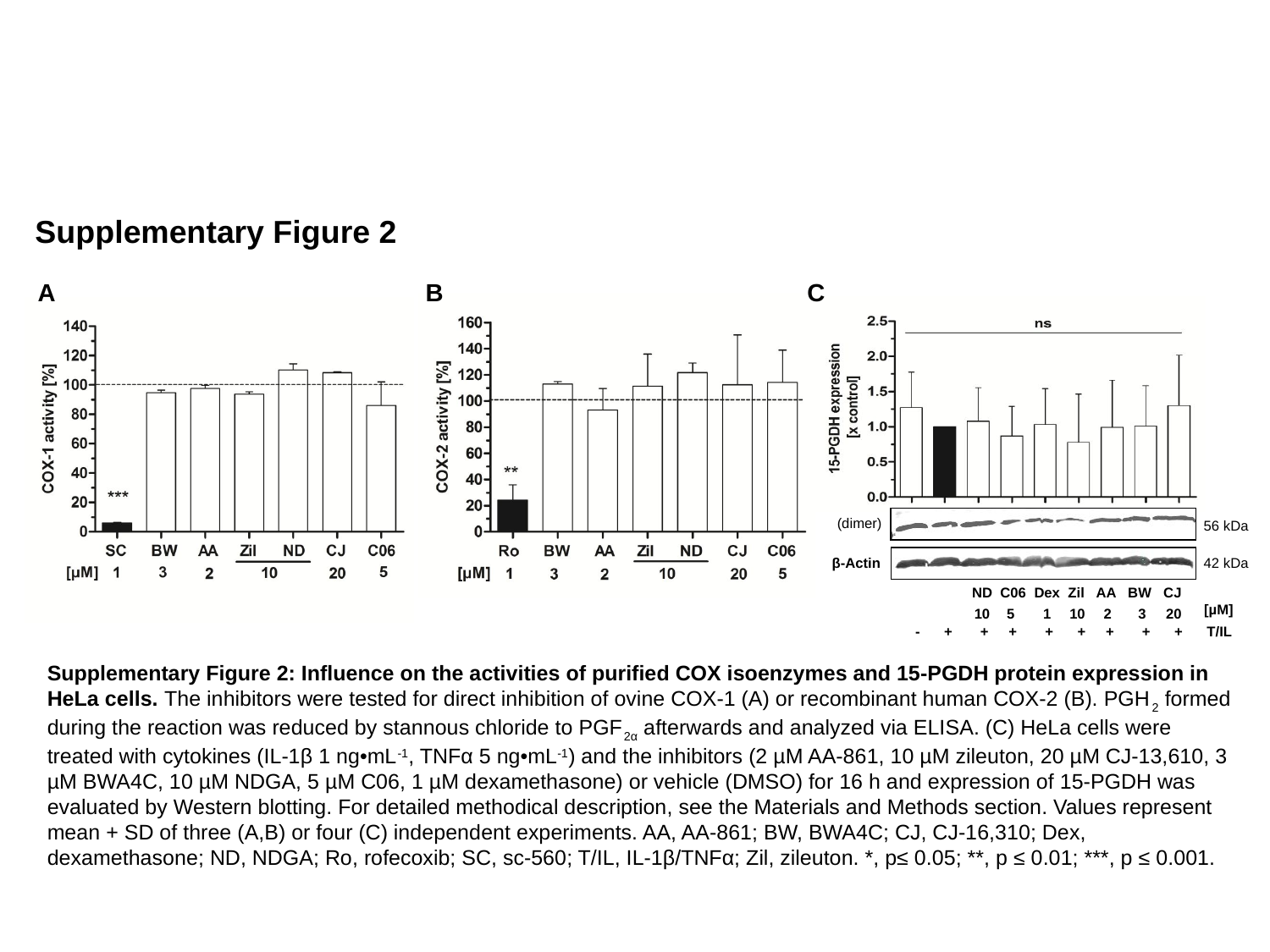

Supplementary Figure 2
 A
 B
 C
(dimer)
 56 kDa
 β-Actin
 42 kDa
 ND C06 Dex Zil AA BW CJ
[µM]
10
5
1
10
2
3
20
- + + + + + + + + T/IL
Supplementary Figure 2: Influence on the activities of purified COX isoenzymes and 15-PGDH protein expression in HeLa cells. The inhibitors were tested for direct inhibition of ovine COX-1 (A) or recombinant human COX-2 (B). PGH2 formed during the reaction was reduced by stannous chloride to PGF2α afterwards and analyzed via ELISA. (C) HeLa cells were treated with cytokines (IL-1β 1 ng•mL-1, TNFα 5 ng•mL-1) and the inhibitors (2 µM AA-861, 10 µM zileuton, 20 µM CJ-13,610, 3 µM BWA4C, 10 µM NDGA, 5 µM C06, 1 µM dexamethasone) or vehicle (DMSO) for 16 h and expression of 15-PGDH was evaluated by Western blotting. For detailed methodical description, see the Materials and Methods section. Values represent mean + SD of three (A,B) or four (C) independent experiments. AA, AA-861; BW, BWA4C; CJ, CJ-16,310; Dex, dexamethasone; ND, NDGA; Ro, rofecoxib; SC, sc-560; T/IL, IL-1β/TNFα; Zil, zileuton. *, p≤ 0.05; **, p ≤ 0.01; ***, p ≤ 0.001.
